# Bispecific antibodies targeting CTLA-4: game-changer troopers in cancer immunotherapy

**DOI:** 10.3389/fimmu.2023.1155778

**Published:** 2023-06-27

**Authors:** Pooya Farhangnia, Shamim Mollazadeh Ghomi, Mahzad Akbarpour, Ali-Akbar Delbandi

**Affiliations:** ^1^ Department of Immunology, School of Medicine, Iran University of Medical Sciences, Tehran, Iran; ^2^ Immunology Board for Transplantation and Cell-Based Therapeutics (ImmunoTACT), Universal Scientific Education and Research Network (USERN), Tehran, Iran; ^3^ Advanced Cellular Therapeutics Facility (ACTF), Hematopoietic Cellular Therapy Program, Section of Hematology & Oncology, Department of Medicine, University of Chicago Medical Center, Chicago, IL, United States; ^4^ Reproductive Sciences and Technology Research Center, Department of Immunology, School of Medicine, Iran University of Medical Sciences, Tehran, Iran

**Keywords:** CTLA-4 (cytotoxic T lymphocyte-associated protein 4), CD152, bispecific antibody (BsAbs), cancer immunotherapy, Bi-specific T cell engagers (BiTEs)

## Abstract

Antibody-based cancer immunotherapy has become a powerful asset in the arsenal against malignancies. In this regard, bispecific antibodies (BsAbs) are a ground-breaking novel approach in the therapy of cancers. Recently, BsAbs have represented a significant advancement in improving clinical outcomes. BsAbs are designed to target two different antigens specifically. Over a hundred various BsAb forms currently exist, and more are constantly being manufactured. An antagonistic regulator of T cell activation is cytotoxic T lymphocyte-associated protein 4 (CTLA-4) or CD152, a second counter-receptor for the B7 family of co-stimulatory molecules was introduced in 1996 by Professor James P. Allison and colleagues. Contrary to the explosive success of dual immune checkpoint blockade for treating cancers, a major hurdle still yet persist is that immune-related adverse events (irAEs) observed by combining immune checkpoint inhibitors (ICIs) or monoclonal antibodies such as ipilimumab (anti-CTLA-4) and nivolumab (anti-PD-1). A promising strategy to overcome this hurdle is using BsAbs. This article will summarize BsAbs targeting CTLA-4, their applications in cancer immunotherapy, and relevant clinical trial advances. We will also discuss the pre-clinical rationale for using these BsAbs, and provide the current landscape of the field.

## 1 Introduction

Numerous advancements in cancer immunotherapy during the last decade have included altering T cell-mediated immunity. Using three kinds of cancer immunotherapies, including immune checkpoint inhibitors (ICIs) or monoclonal antibodies (mAbs), genetically modified T cells expressing chimeric antigen receptors (CARs), and bispecific antibodies (BsAbs), are now licensed for clinical use (1, 2). mAb-based cancer immunotherapy has become a potent weapon in the arsenal against malignancies. Up to now, the FDA has approved several therapeutic mAbs for treating various malignancies and autoimmune disorders. Target cells may undergo cellular cytotoxicity when mAbs selectively attach to their antigens. After gaining crowning achievements of mAbs in treating cancer, another molecular platform of mABs was developed. Meanwhile, BsAbs, which target two antigens simultaneously, were introduced as therapeutic medications for various cancers (3).

During the 1960s, Nisonoff and colleagues played a significant role in popularizing the concept of BsAbs. The subsequent development of innovative antibody engineering methods produced numerous BsAb molecular platforms. BsAbs are superior to mAbs in several ways. Outstanding cytotoxicity effects of BsAbs in cancers are one of these benefits. BsAbs also exhibit a lower level of therapeutic resistance (4). Some BsAbs have a small size, typically consisting of two basic single‐chain fragments with variable domain (5). As an example, BsAbs that are based on single-chain fragment variables (scFvs) have demonstrated a high degree of specificity for tumor cells and are able to penetrate tissues effectively (6). BsAbs may be an innovative new pillar in the fight against cancer. In terms of improving clinical treatment results, BsAbs represent a promising milestone. Many BsAbs have been developed within the context of tumor immunotherapy throughout the last few decades, and the first BsAb named blinatumomab was licensed in 2009 (7, 8). Lymphomas, in particular, appear to respond well to BsAbs, while myeloid neoplasias and solid tumors have shown more limited success (9). Although BsAbs have been shown to penetrate solid tumors effectively, there are concerns about their safety due to their short half-life, and this remains an area of active research (10).

An antagonistic regulator of T cell activation is cytotoxic T lymphocyte-associated protein 4 (CTLA-4) or CD152, a second counter-receptor for the B7 family of co-stimulatory molecules, which was introduced in 1996 by professor James Allison and colleagues (11). CTLA-4 is an inhibitory immune checkpoint, which inhibits immune responses through both an intrinsic mechanism that transmits a negative signal directly to effector T lymphocytes (T_eff_) and an extrinsic mechanism that is primarily connected to regulatory T lymphocyte (T_reg_) functions (12). B7-1 (CD80) and B7-2 (CD86) are the ligands shared by CD28 and CTLA-4, which have a greater affinity to bind CTLA-4. Consequently, CTLA-4 and CD28 are antagonistic concerning ligand binding (12, 13). The discovery that CTLA-4 can impede T cell activation provided the basis for the notion that interrupting its function might allow T cells to mount a therapeutic attack on cancer (14, 15). In mice, mAbs against CTLA-4 improved the immune system’s ability to fight against colon cancer and fibrosarcoma (11). Moreover, in subsequent exposure to tumor cells, animals that had been given anti-CTLA-4 treatment were able to quickly eradicate the cancerous cells by means of the immune system. This suggests that suppressing CTLA-4 leads to sustained immunological memory (11, 16).

At present, numerous clinical trials are underway to investigate the effectiveness of BsAbs targeting CTLA-4 in treating various types of cancer. These trials have yielded promising results in certain types of tumor cells and have been associated with a prolonged anti-tumoral response (17–19). Although BsAbs targeting CTLA-4 have been a significant breakthrough in the field of cancer immunotherapy, there are still numerous aspects that require clarification and challenges that need to be addressed related to the safety, effectiveness, and range of tumors that could be treated. This review summarizes BsAbs targeting CTLA-4, their applications in cancer immunotherapy, relevant challenges, and clinical trial advances. We also discuss the pre-clinical rationale for using BsAbs targeting CTLA-4 and provide the current landscape of the field.

## 2 Bispecific antibodies mechanisms of action: a general viewpoint

**Figure 1:**
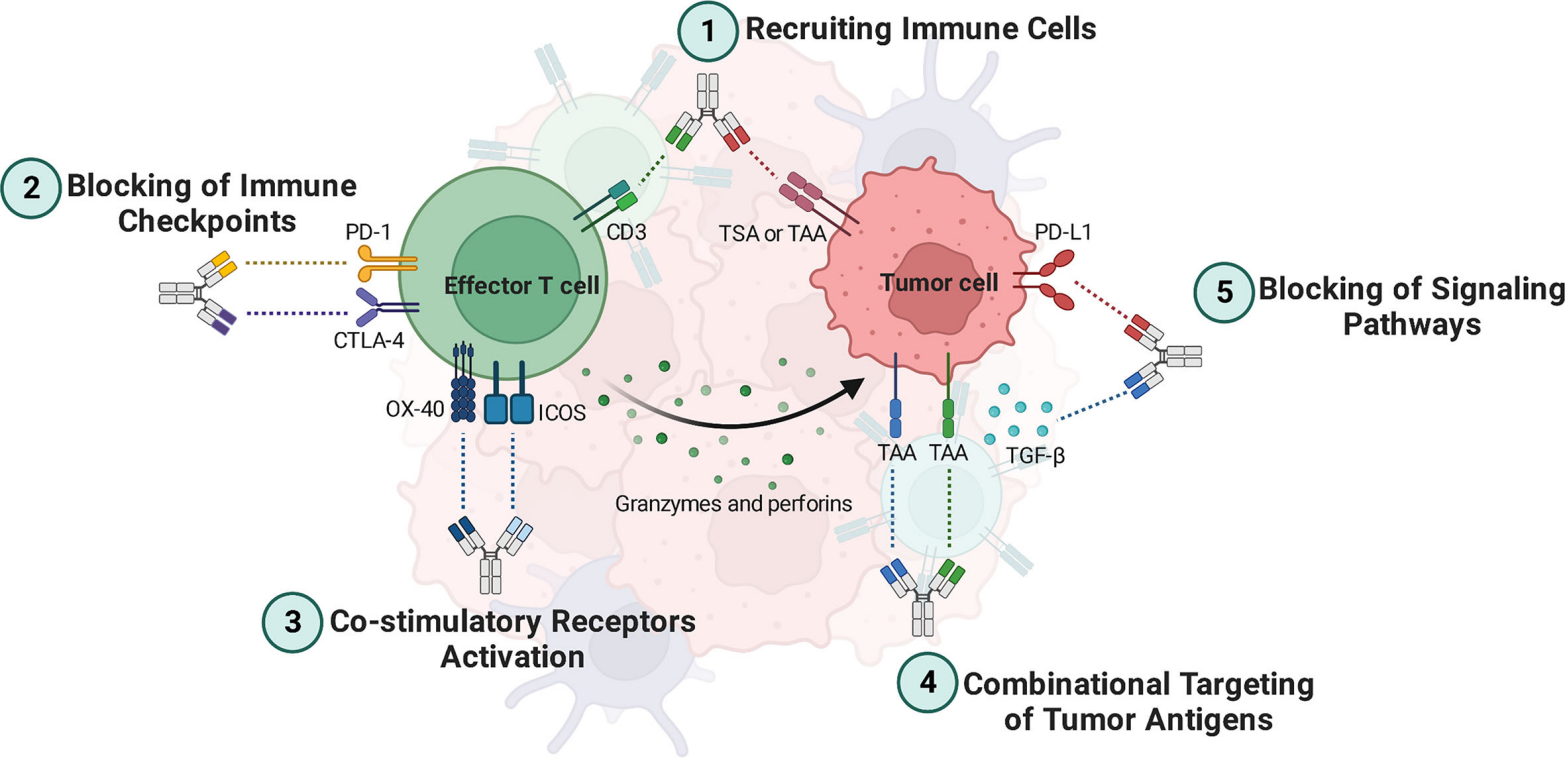
Bispecific antibodies (BsAbs) mechanisms of action. **1)** BsAbs can guide effector T cells toward cancer cells, where they can lead to lysis them. **2)** Simultaneous inhibition of two inhibitory immune checkpoints leads to more reinvigoration of effector T cells to eradicate the tumor cells. **3)** BsAbs can be deployed as agonistic agents activating co-stimulatory receptors such as ICOS and OX-40, leading to better activation of tumor-specific effector T cells. **4)** BsAbs targeting dual TAAs bind specifically to tumor cells that express both antigens. Thus, tumor cells can be targeted more specifically. **5)** BsAbs can suppress signaling pathways that are involved in angiogenesis and T cell exhaustion, such as those that inhibit TGF-β and PD-L1 signaling pathways. TAA, Tumor-associated antigen; TSA, Tumor-specific antigen; ICOS, Inducible T cell co-stimulator; TGF-β, Transforming growth factor-beta; PD-1, Programmed cell death protein 1; PD-L1, Programmed death-ligand 1; CTLA-4, Cytotoxic T-lymphocyte associated protein 4.

BsAbs are designed to bind two distinct antigens concurrently. BsAbs exert their functions by redirecting and activating immune cells, inhibiting immune cell co-inhibitory receptors, activating co-stimulatory molecules, inhibiting signaling pathways, and combinatorial targeting of cancer antigens (**Figure 1**) (20).

**Recruiting Immune Cells.** In addition to stimulating T cells, BsAbs such as blinatumomab and catumaxomab that target CD3, CD19, and EpCAM also guide these cells toward cancer cells, where they can lead to lysis them (4). Besides, BsAbs such as AFM13 can crosslink the cancer antigen CD30 on tumor cells with CD16a on natural killer (NK) cells, rerouting NK cells to the tumor cells for lysing by antibody-dependent cellular cytotoxicity (ADCC) (3).

**Blocking of Immune Checkpoints and Co-inhibitory Molecules.** Programmed death-ligand 1 (PD-L1), programmed cell death protein 1 (PD-1), and CTLA-4 are well-known immune checkpoints that prevent immune cells from becoming activated. Thus, blocking immune checkpoints with antibodies can reinvigorate immune cells. BsAbs targeting immune checkpoints are mostly used for treating solid tumors, including AK104 (PD-1×CTLA-4) (17), MGD019 (PD-1×CTLA-4) (18), XmAb20717 (PD-1×CTLA-4) (21), MEDI5752 (PD-1×CTLA-4) (22), MGD013 (PD-1×Lymphocyte-activation gene 3 [LAG-3]) (23), and RO7121661 (PD-1×T-cell immunoglobulin and mucin domain 3 [TIM-3]) (NCT03708328), which are capable of targeting two immune checkpoints simultaneously. Dysregulation of lymphocyte functions co-expressed by PD-1 and LAG-3 is common, and combined therapy involving PD-1 and LAG-3 can effectively restore T cell function (24). Furthermore, BsAbs targeting immune checkpoints combined with tumor-associated antigens (TAAs) have been developed, including AK112 (PD-1×Vascular endothelial growth factor [VEGF]) and IBI315 (PD-1×Human epidermal growth factor receptor 2 [HER2]) (4, 25).

**Co-stimulatory Receptors Activation.** To maintain a stable anti-cancer immune response against advanced malignancies, one strategy might be proposed that co-stimulatory receptors can be targeted along with the blockage of immunological checkpoints. Numerous BsAbs have been emerging for binding co-stimulatory receptors, such as inducible T cell co-stimulator (ICOS) or CD278, OX40, and 4–1BB (3, 20). OX40, like CTLA-4, is significantly up-regulated on activated T cells in the tumor microenvironment (TME), particularly on Tregs (26, 27). Targeting two overexpressed receptors, for instance CTLA-4 and OX40, in the tumor has the potential to increase the localization of BsAbs to the tumor site compared to monospecific antibodies, which may reduce the risk of systemic T cell activation and improve efficacy. Additionally, it has been suggested that combining a checkpoint inhibitor with a T cell co-stimulatory agonistic antibody may convert a “cold” tumor into a “hot” tumor by enhancing T cell expansion and effector functions while controlling the suppressive function of Tregs (28, 29).

**Combinational Targeting of Tumor Antigens.** “On-target off-tumor” toxicity is a significant issue that needs to be considered in antibody-based therapeutic modalities. This toxicity refers to a side effect of cancer immunotherapy where the immune system attacks not only cancer cells but also healthy cells expressing the same target antigen, leading to unintended damage to healthy tissues (30). In line with this issue, both healthy and malignant cells express TAAs. One of the BsAb’s advantages is that it targets tumor cells more precisely, which directs its power to only tumor cells in circulation, TME, and metastatic sites and not healthy cells. Thus, healthy cells are unharmed as a result of this. In other words, BsAbs targeting dual TAAs bind specifically to cancer cells that express both antigens, while avoiding healthy cells that express only one antigen (31).

The fact that many cancer antigens are cleaved by enzymes, releasing soluble extracellular domains, presents another obstacle. Indeed, a major challenge in developing immunotherapies for hematologic malignancies, for instance, acute lymphocytic leukemia (ALL), is that cancer cells can lose the CD19 antigen while retaining CD22 after CD19 shedding (32). Due to this mechanism, tumor antigen escape happens, but BsAbs can offset this mechanism *via* combinational targeting. As an illustration, a BsAb called DT2219ARL has been developed to overcome antigen loss-mediated relapse in ALL. DT2219ARL targets CD19 and CD22, which are two antigens commonly expressed in ALL cells (20, 33, 34).

**Blocking of Signaling Pathways.** BsAbs can reduce the progression of tumors by targeting the molecules of signaling pathways involved in angiogenesis, metastasis, and proliferation. In this regard, there are several BsAbs such as YM101 (35–37), MCLA-128 (zenocutuzumab) (38, 39), BI 836880 (40, 41), vanucizumab (42), ABT-165 (43), OMP-305B83 (navixizumab) (44, 45), TR009 (ABL001) (46), and EMB01 (47, 48). For instance, YM101 has the ability to specifically bind to transforming growth factor-beta (TGF-β) and PD-L1. Results from *in vitro* experiments demonstrated that YM101 effectively inhibited the biological impacts of TGF-β and the PD-1/PD-L1 pathways, which includes the activation of Smad signaling, induction of epithelial-mesenchymal transition, and immunosuppressive activities (35). Furthermore, MCLA-128 targets VEGF and angiopoietin-2 (4).

## 3 Mechanism of action of bispecific anti-CTLA-4 antibodies

BsAbs that target CTLA-4 are a novel class of immunotherapies that are being developed to improve the effectiveness and safety of immune checkpoint blockade in cancer treatment. The mechanism of action of bispecific anti-CTLA-4 antibodies involves, for instance, the simultaneous binding of CTLA-4 on T_regs_ and co-stimulatory receptor such as OX-40 on T_eff_ (49). The dual engagement of these two molecules helps to modulate the balance between immune activation and suppression, which is important for achieving an optimal anti-tumor response. For instance, by binding to CTLA-4 on T_regs_, ATOR-1015 can selectively deplete or down-regulate these cells (49), which are known to play a role in suppressing immune responses and promoting tumor growth. At the same time, by binding to OX-40 on T_eff_, ATOR-1015 can enhance T cell activation and proliferation, leading to increased colon, pancreatic, and bladder tumor cell killing (49, 50).

BsAbs targeting both CTLA-4 and another molecule, such as PD-1 (17, 51) or PD-L1 (52), enhance the immune response against cancer by blocking two separate immune checkpoints that can inhibit anti-tumor immunity. By targeting both CTLA-4 and PD-1, these bispecific antibodies can simultaneously promote activation and proliferation of T cells, reduce regulatory T cell function, and enhance the killing of tumor cells by cytotoxic T lymphocytes. The combination of these effects allows for a more robust and sustained anti-tumor response (4, 20).

## 4 Anti-CTLA-4 antibody and combinational therapies

There is a compelling strategy regarding combinational using the anti-CTLA-4 mAbs with other therapies against poorly immunogenic tumors (53). Wei et al. (54) demonstrated that contrary to either monotherapy, CTLA-4/PD-1 dual immune checkpoint blockade is sufficient to stimulate specific cellular responses. Anti-CTLA-4 and anti-PD-1 inhibition considerably improves responses in pre-clinical tumor models, leading to much higher T_eff_-to-suppressor cells (myeloid-derived suppressor cell and T_reg_) ratios and the generation of pro-inflammatory cytokines including interferon-gamma (IFN-γ) and tumor necrosis factor-alpha (TNF-α) (55). Combinational therapy ipilimumab plus nivolumab led to enhance the effectiveness of treatment in melanoma (56), renal cell carcinoma (57), colorectal cancer (58) compared with monotherapy. In melanoma, the combination of PD-1 and CTLA-4 blockade was linked to specific cellular immune responses, including a dramatic increase in cytokine production, an increase in T cell frequency, and a decrease in circulating B cells (59, 60).

## 5 Pre-clinical rationale for using bispecific antibodies targeting CTLA-4

The currently known BsAbs are fragment-based, symmetric, asymmetric or Bi-specific T cell engagers (BiTEs). These forms determine their half-life, immunogenicity, target selectivity, and production complexity (61). Contrary to the explosive success of dual immune checkpoint blockade for treating cancers, two major hurdles persist. First, immune-related adverse events (irAEs) were observed by combination ICIs such as ipilimumab and nivolumab (62). Second, compared to BsAbs, there is an MHC restriction of the TCR when using ICIs, leading to immune escape (63). A promising strategy to overcome these hurdles is simultaneously co-blockade of two cancer antigens, for example, CTLA-4 and PD-1, preferentially in the TME by BsAbs, restricting immune responses specific to the tumor site. Thus, it can be expected that immune-mediated toxicity toward normal tissues is reduced (12).

Immunotherapies for cancer, such as the blockade of CTLA-4 and PD-1, often result in severe autoimmunity as a side effect (64, 65). Overall, there is a tendency for the efficacy of anti-tumor responses to be associated with the occurrence of autoimmune diseases, particularly when a systemic approach is taken to deplete T_regs_ (66). A promising strategy to elicit potent anti-tumor immune responses while minimizing the risk of inducing detrimental autoimmune reactions might be to selectively engage effector T_regs_ in the TME using BsAbs. This approach has the potential to preserve the pool of naive T_regs_ in non-tumor tissues, which are essential for maintaining immune tolerance and preventing autoimmunity. In other words, it is desirable to avoid T_reg_ depletion in normal tissues while targeting immune checkpoint pathways in the TME. One potential advantage of using BsAbs is that they can be designed to selectively bind to cells expressing both PD-1 and CTLA-4, which are mainly tumor-infiltrating lymphocytes (TILs). Thus, by selectively targeting TILs, BsAbs may spare T_regs_ in normal tissues and avoid the potential adverse effects associated with their systemic depletion.

On the one hand, the possibility that anti-tumor reactive T cells will be inadequate in quantity or malfunctioning and anergic are two key issues with chimeric antigen receptor (CAR) T cell treatment that are addressed by BsAbs. On the other hand, increased tumor-reactive T cell frequency is the ultimate goal of BsAbs. In order to accomplish this, T cells and tumor cells are connected effectively *via* BsAbs in an intercellular space name immunological synapse. Their activation occurs without the need for co-stimulation, peptide antigen presentation, and major histocompatibility complex (MHC) class 1/2 (61, 67). All in all, the aforementioned basic concepts provide a rationale for using BsAbs.

There are some BsAbs, which have been described in pre-clinical (**Table 1**) and clinical investigation (**Table 2**). These include XmAb20717 (Vudalimab), MEDI5752, AK104, MGD019, KN046, and ATOR-1015. According to preliminary results, XmAb20717, which targets CTLA-4 and PD-1 simultaneously, was well-tolerated in patients with advanced cancer and had complete and partial responses in various tumor types. T cell population changes in the tumor and surrounding tissues were consistent with effective dual checkpoint inhibition (21). In pre-clinical studies, using another BsAb targeting CTLA-4 and PD-1 named MEDI5752 was associated with lower cytotoxicity and equivalent activity to anti-PD-1 and anti-CTLA-4 antibodies, providing an improved therapeutic index (22). In the following, we will elaborate on the clinical trials of BsAbs targeting CTLA-4 (**Table 2**).

**Table 1:** Pre-clinical status of BsAbs targeting CTLA-4.

Name	Targets	Main findings	Reference
MEDI5752	CTLA-4 + PD-1	1. High saturation of CTLA-4 on PD-1^+^ tumor cells by MEDI5752. 2. Inhibition of CTLA-4 on TILs while sparing peripheral T cell populations and reducing toxicity. 3. MEDI5752 induces internalisation and subsequent degradation of PD-1 by tethering CTLA-4 to PD-1.	(22)
MGD019	CTLA-4 + PD-1	1. Combinatorial blockade of PD-1 and CTLA-4 *via* single molecule. 2. MGD019 is well-tolerated in non-human primates. 3. Increasing in the count of Ki67^+^CD8^+^ and ICOS^+^CD4^+^ T cells upon MGD019 administration.	(18)
ATOR-1015	CTLA-4 + OX40	1. ATOR-1015 activates T cells and reduces Tregs *in vitro*. 2. In various preclinical models, ATOR-1015 reduces tumor growth and improves survival, including in bladder, colon, and pancreas cancer models. 3. ATOR-1015 generates long-term tumor-specific immunological memory and enhances response to PD-1 inhibition. 4. ATOR-1015 targets the tumor area, where it boosts the number and activation of CD8^+^ T-cells and decreases Tregs.	(49)

CTLA-4, Cytotoxic T-lymphocyte-associated protein 4; PD-1, Programmed cell death protein 1; Treg, Regulatory T cell; TIL, Tumor-infiltrating lymphocyte; ICOS, Inducible T-cell costimulator.

**Table 2:** BsAbs targeting CTLA-4 in clinical development.

Name	Type and Structure	Targets	Indication	Treatment regimen	Status/Phase	Participants	Developed by	NCT identifier
XmAb20717 (Vudalimab)	Humanized BsAb/XmAb technology	CTLA-4 + PD-1	-Melanoma -Breast carcinoma -HCC -Renal cell carcinoma -Colorectal carcinoma -non-small-cell lung carcinoma - Cervical cancer -Mesothelioma	MT	Active, not recruiting/I	154	Xencor	NCT03517488
MEDI5752	Fully human BsAb (a monovalent bispecific human IgG_1_ mAb with an engineered Fc domain)	CTLA-4 + PD-1	Advanced solid tumors	Combinational therapy: Pemetrexed, Carboplatin, Pembrolizumab, Paclitaxel	Recruiting/I	366	MedImmune	NCT03530397
			Renal cell carcinoma	Combinational therapy: Axitinib, Lenvatinib	Recruiting/I	70	MedImmune	NCT04522323
			Metastatic NSCLC	Combinational therapy: Durvalumab, Danvatirsen, Oleclumab, Pemetrexed, Carboplatin, Gemcitabine, Cisplatin, Nab-paclitaxel, AZD2936	Active, not recruiting/I	258	AstraZeneca	NCT03819465
AK104 (Cadonilimab)	IgG_1_ scaffold Fc-engineered humanized antibody	CTLA-4 + PD-1	Cervical cancer	MT	Completed/II	30	Akeso	NCT04380805
			Recurrent or metastatic cervical cancer	Combinational therapy: Bevacizumab, Paclitaxel, Cisplatin or Carboplatin	Active, not recruiting/II	50	Akeso	NCT04868708
			Nasopharyngeal carcinoma	MT	Completed/II	34	Akeso	NCT04220307
			Advanced MSI-H/dMMR gastric carcinoma and colorectal cancer	MT	Not yet recruiting/II	29	Peking University	NCT04556253
			-Gastric adenocarcinoma -Advanced solid tumors -Gastroesophageal Junction Adenocarcinoma	Combinational therapy: Oxaliplatin, Capecitabine	Active, not recruiting/I,II	338	Akeso	NCT03852251
			HCC	Combinational therapy: Lenvatinib	Active, not recruiting/II	32	Akeso	NCT04728321
			Liver cancer	Combinational therapy: Lenvatinib	Recruiting/I,II	30	Akeso	NCT04444167
			NSCLC	Combinational therapy: Anlotinib	Active, not recruiting/I,II	114	Akeso	NCT04646330
			NSCLC	Combinational therapy: Anlotinib	Not yet recruiting/II	30	Chinese PLA General Hospital	NCT04544644
			Advanced or metastatic solid tumors	Combinational therapy: AK119 (anti-CD73)	Recruiting/I	195	Akeso	NCT04572152
MGD019	a tetravalent bispecific (2 × 2) Fc-bearing based on DART platform	CTLA-4 + PD-1	-NSCLC -Prostate cancer metastatic -Cutaneous melanoma -Colorectal cancer	Combinational therapy: Lorigerlimab	Active, not recruiting/I	287	MacroGenics	NCT03761017
KN046	Humanized bispecific single domain Fc fusion protein antibody	CTLA-4 + PD-L1	NSCLC	Combinational therapy: Paclitaxel, Pemetrexed, Carboplatin	Unknown/II	50	Jiangsu Alphamab Biopharmaceuticals	NCT04054531
			Stage IV NSCLC	MT	Unknown/II	149	Jiangsu Alphamab Biopharmaceuticals	NCT03838848
			HER2 positive solid tumor	Combinational therapy: KN026 (anti-HER2)	Recruiting/I	24	Peking University	NCT04040699
			TNBC	Combinational therapy: Nab-paclitaxel	Active, not recruiting/I,II	52	Jiangsu Alphamab Biopharmaceuticals	NCT03872791
			Esophageal squamous cell carcinoma	MT	Completed/II	45	Jiangsu Alphamab Biopharmaceuticals	NCT03925870
			Advanced gastrointestinal tumors	Combinational therapy: Donafenib Tosilate	Recruiting/I, II	42	Suzhou Zelgen Biopharmaceuticals	NCT04612712
			HCC	Combinational therapy: Lenvatinib	Recruiting/II	55	Peking University Cancer Hospital & Institute	NCT04542837
			Locally advanced and metastatic pancreatic cancer	Combinational therapy: Gemcitabine, Albumin-Paclitaxel, Oxaliplatin, Irinotecan, Leucovorin, Fluorouracil	Recruiting/I,II	60	Changhai Hospital	NCT04324307
			Squamous NSCLC	MT	Active, not recruiting/III	482	Jiangsu Alphamab Biopharmaceuticals	NCT04474119
			Thymic carcinoma	MT	Recruiting/II	29	Weill Medical College of Cornell University	NCT04925947
			Thymic carcinoma	MT	Recruiting/II	66	Jiangsu Alphamab Biopharmaceuticals	NCT04469725
ATOR-1015	Human IgG_1_ BsAb	CTLA-4 + OX40	-Solid Tumor -Neoplasms	MT	Completed/I	33	Alligator Bioscience AB	NCT03782467

mAb, Monoclonal antibody; BsAb, Bispecific antibody; Fc, Fragment crystallizable region; CTLA-4, T-lymphocyte-associated protein 4; PD-1, Programmed cell death protein 1; NSCLC, Non-small cell lung cancer; HCC, Hepatocellular carcinoma; TNBC, Triple-negative breast cancer; MT, Monotherapy; DART, Dual affinity retargeting.

## 6 Current landscape and clinical trials

**Figure 2:**
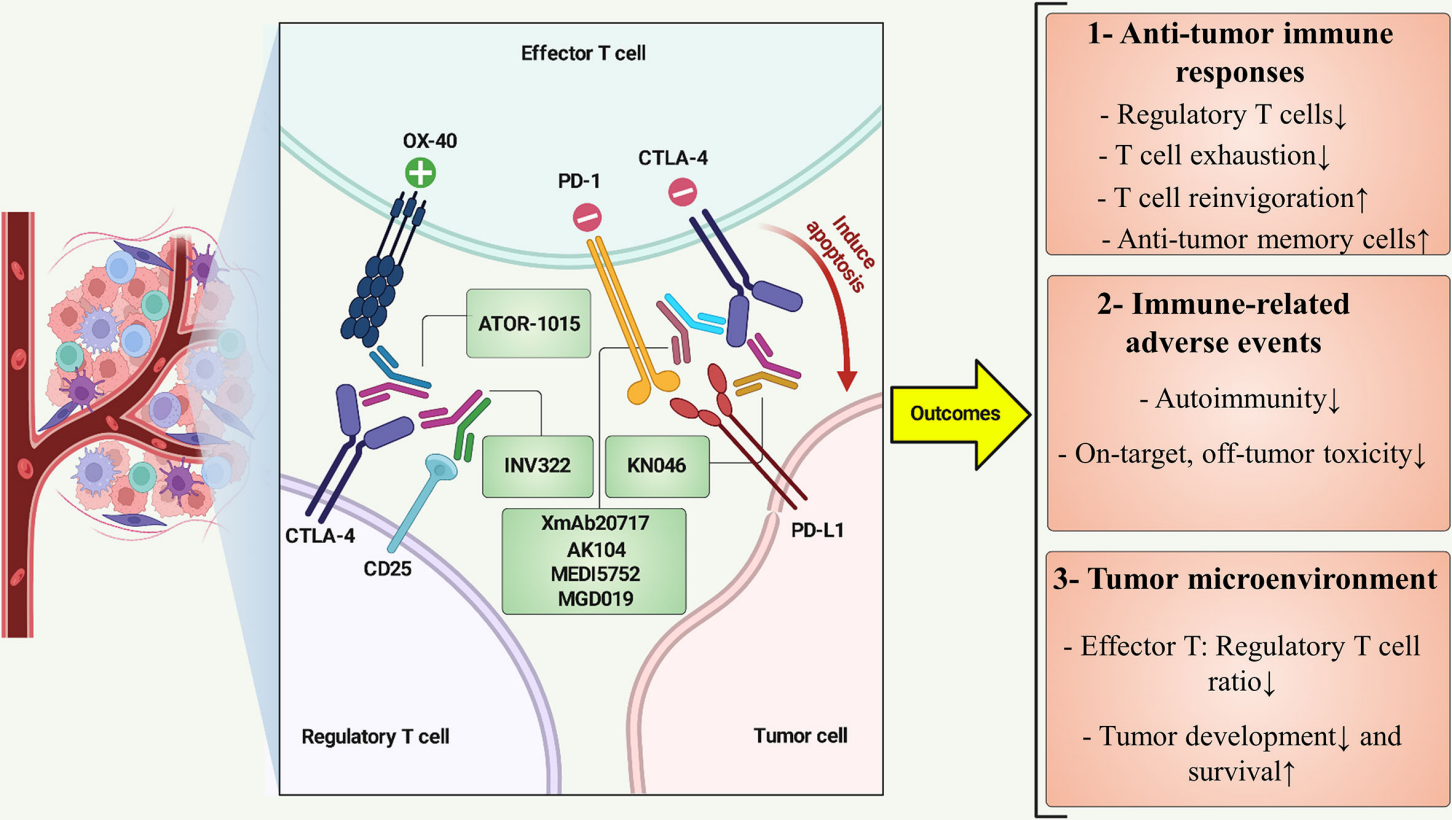
BsAbs targeting CTLA-4 at a view of cross-talk between immune and cancer cells. ATOR-1015 can simultaneously target CTLA-4 and OX-40 on regulatory T cell and effector T cell, respectively, resulting in effector T-cell stimulation and improved cancer immunity. INV322 binds to CD25 and CTLA-4, which restricts the function of regulatory T cells in the tumor microenvironment. CTLA-4 and PD-L1 are recognized by KN046, eventually preventing T-cell exhaustion and inducing apoptosis in tumor cells. BsAbs such as XmAb20717, AK104, MEDI5752, and MGD019 inhibit PD-1 and CTLA-4, revitalizing tumor-specific cytotoxic T lymphocytes. Positive and negative symbols indicate stimulatory and inhibitory role of markers, respectively. ↑, Increase; ↓, Decrease.

The landscape of clinical anti-CTLA-4 BsAbs immunotherapy continues to progress promptly. Recently, there have been numerous illustrations of these BsAbs in clinical and pre-clinical phases. Rudimentary findings revealed positive clinical outcomes, which can be classified into three categories, including i) Anti-tumor immune responses, ii) Immune-related toxicities, and iii) TME (**Figure 2**). The following sections summarize the current landscape and the clinical trials.

### 6.1 CTLA-4×PD-1 BsAbs

**XmAb20717.** XmAb20717 is a heterodimeric antibody composed of two different protein subunits, designed with a modified Fc domain that prevents interactions with Fc-gamma receptor (FcγR). Additionally, the incorporation of Xtend^™^ technology enhances its pharmacokinetic properties to promote a longer half-life (21). A human clinical trial is investigating the effects of XmAb20717 (NCT03517488). Patients with advanced solid tumors, including melanoma, renal cell carcinoma, non-small cell lung cancer (NSCLC), castration-resistant prostate cancer, are enrolled in phase 1 multiple doses, ascending dose escalation clinical trial to determine the pharmacokinetic (PK), maximum-tolerated dose, safety, and tolerability.

**MEDI5752.** MEDI5752 is a fully human monovalent bispecific human IgG1 mAb with an engineered Fc domain designed to suppress the PD-1 pathway and provide regulated CTLA-4 inhibition, favoring greater blocking of PD-1^+^ activated T cells. The variable domains of an anti-PD-1 mAb and tremelimumab (an anti-CTLA-4 mAb) were combined to generate this BsAb. The engineering of the IgG1 constant heavy chains also diminished the Fc-mediated immune effector functions. The higher anti-tumor activity was stimulated by MEDI5752, which was found to accelerate PD-1 internalization and degradation and to accumulate preferentially in tumors. MEDI5752 could fully saturate CTLA-4 on cells expressing PD-1 and CTLA-4 at lower doses than those needed to fully saturate CTLA-4 on cells that did not express PD-1 (51). A phase 1, first-time-in-human, multicenter, open-label, dose-escalation and dose-expansion clinical trial was designed to evaluate the safety, tolerability, efficacy, PK, and immunogenicity of MEDI5752 in participants with advanced solid tumors, when administered as a single agent or combined with chemotherapeutic drugs (NCT03530397).

**AK104.** PD-1 and CTLA-4 are concurrently targeted by the humanized IgG1 tetrameric BsAb known as AK104. Indeed, AK104 is an IgG_1_ scaffold Fc-engineered humanized antibody. Early research revealed that AK104 has promising anti-tumor effectiveness in liver cancer and a better safety profile than co-administering anti-PD-1 and anti-CTLA-4 mAbs (17). A phase 2, global, multicenter, open-label, single-arm study evaluated the efficacy, safety, tolerability, PK, and immunogenicity of AK104 monotherapy in adult subjects with previously treated recurrent or metastatic cervical carcinoma (NCT04380805).

**MGD019.** Berezhnoy et al. (18) have developed a BsAb in the IgG4 isotype known as MGD019 using a dual-affinity re-targeting antibody (DART) platform. This BsAb consists of an engineered tetravalent CTLA-4/PD-1 molecule. Two variable fragments with their variable heavy chain components switch to form a DART molecule (68). The PD-1 and CTLA-4 binding domains are derived from retifanlimab and human mAb 4B6, respectively (18). Compared to bi-single domain antibodies, this novel structure enables greater conformational flexibility during antigen-antibody recognition (18). To prevent the potential depletion of T_eff_ and T_regs_, MGD019 carries Fc mutations, which cause the lack of Fc-mediated effector function and limit the ability to trigger ADCC. Innovative approaches include modulating the Fc effector domain or directing the immune response preferentially toward TME, which may improve therapy efficacy and minimize immune-mediated damage (27, 69). Despite increasing human T cell activation *in vitro*, MGD019 did not exhibit any Fc-mediated effector activity. MGD019 showed promising therapeutic efficacy with acceptable safety in patients with advanced solid tumor cancer, including ovarian, breast, lung, colon cancer, who had had much pre-treatment and was well-tolerated in cynomolgus monkeys. Notably, in animals treated with MGD019, no alterations were observed in either the tissue-resident or circulating T_reg_ populations (18).

### 6.2 CTLA-4×PD-L1 BsAb

**KN046.** KN046 (also known as alphamab), a unique humanized bispecific single domain Fc fusion protein antibody, can simultaneously inhibit the PD-1/PD-L1 pathway and the CTLA-4 pathway. Thus, it reinvigorates the immunological responses of exhausted T lymphocytes to the tumor. The findings of both pre-clinical and clinical testing with KN046 have shown good effectiveness, accompanied by decreased levels of systemic toxicity. KN046 is now being evaluated in several clinical studies in phase 1 and 2 as either a single agent or as part of combination regimens in tumor types and stages (52, 70). In patients with HER2-positive gastrointestinal tumors, preliminary efficacy and safety results of KN026 (a BsAb targeting two distinct HER2 epitopes) and KN046 have been reported. In both treatment-naive and extensively pretreated HER2-positive gastrointestinal cancers, KN026 combined with KN046 demonstrated the potential to improve clinical benefit to the current standard of care (71). A phase 2 clinical trial of KN046 was investigated in patients with metastatic NSCLC who failed first-line treatment. Results showed that KN046 was a successful second-line therapy for advanced NSCLC and was well-tolerated. In both squamous and non-squamous NSCLC, KN046 demonstrated a potential overall survival improvement (19). KN046 and Nab-paclitaxel showed safety, tolerability, and efficacy results in patients with metastatic triple-negative breast cancer (72).

### 6.3 CTLA-4×OX40 BsAb

**ATOR-1015.** An improved form of the Ig-like V-type domain of human CD86 was linked to an agonistic OX40 antibody, and eventually, a BsAb in the IgG1 form known as ATOR-1015 was generated. This BsAb simultaneously targets CTLA-4 and OX40. OX40, or CD134, is a powerful immunological co-stimulatory molecule that can be induced on activated CD4^+^ and CD8^+^ T cells. *In vitro*, ATOR-1015 stimulated T lymphocyte activation and T_reg_ reduction. Many syngeneic tumor models, such as those of bladder, colon, and pancreatic cancer, responded well to treatment with ATOR-1015, which slowed tumor development and increased survival. It is further shown that ATOR-1015 increases the response to PD-1 blockade and generates tumor-specific and long-term immunological memory. Additionally, ATOR-1015 is localized to the tumor site, enhancing the frequency and activation of CD8^+^ T lymphocytes while decreasing the frequency of T_regs_ (49). A phase 1 clinical trial investigated the safety and tolerability of ATOR-1015 when administered as repeated intravenous infusions to patients with advanced and refractory solid malignancies (NCT03782467).

### 6.4 CTLA-4×GITR BsAb

A receptor called glucocorticoid-induced TNFR family-related gene (GITR) promotes T lymphocyte activation against tumor cells. A patent known as WO2018091739 employs a BsAb targeting CTLA-4 and GITR to treat colon carcinoma. This BsAb has a basic structure that includes two binding sites for GITR, which are formed by the antigen-binding sites of both the heavy and light chains. In addition, there are two binding sites for CTLA-4, which are created by a CTLA-4 binding domain that is connected to the kappa constant region of the light chain through a linker at the carboxyl end (73). BsAb triggered T_eff_ and tumor inhibition in colon carcinoma-bearing mice (73). However, no clinical studies still demonstrate using this BsAb to treat cancer patients.

### 6.5 CTLA-4×CD25 BsAb

In the clinic, targeting T_regs_ has shown promise, but current strategies are constrained by on-target, off-tumor stimulation of autoimmune-related toxicities linked to global T_reg_ blocking. To limit these toxicities, a BsAb known as INV322 was engineered to engage the Tregs of TME preferentially. It is created using Invenra’s B-Body^®^ platform, which is fully human in origin, and has a wild-type IgG_1_ structure that enables Fc-gamma-mediated effector function (74). INV322 targets CD25 and CTLA-4 on T_regs_ with lower-affinity monovalent interactions, supporting the selective blockade of tumor-restricted T_regs_ function and depletion by Fc-mediated clearance. *In vivo*, the development of anti-tumor memory cells, tumor-protective activity, T_reg_ depletion, and a rise in the T_eff_: T_reg_ ratio inside the TME were associated with INV323 treatment (74).

## 7 Conclusion, challenges, and future directions

Over the past three decades, there has been a significant evolution from the simple development and modification of mAbs without any further engineering to more complex antibody derivatives in a wide range of shapes and sizes, particularly BsAbs (75). Researchers’ interest in BsAb technology has grown significantly over time due to its outstanding potential for clinical applications. This development has established a strong basis for BsAb-based cancer immunotherapy. BsAbs are a novel approach to the fight against cancer. Overall, BsAbs have represented a significant advancement in improving clinical results. BsAbs might play a fascinating function in treating cancer. IrAEs caused by combining ICIs like ipilimumab and nivolumab remain a significant obstacle despite the tremendous success of treating cancers. BsAbs are a viable strategy to overcome this obstacle because the simultaneous co-blockade of two cancer antigens, such as CTLA-4 and PD-1, primarily in the TME by BsAbs may restrict immune responses, particularly to the tumor site. Thus, healthy cells and tissues are not damaged.

Although BsAbs are well positioned as a safe immunotherapy, outstanding questions remain open. Elucidating the significant parameters that determine anti-CTLA-4 BsAbs potency and persistence will be essential as the field progresses to evolving strategies to address obstacles specific to each type of cancer. Remarkably, as the field of cancer immunotherapy, particularly BsAbs, continues to innovate rapidly, it is important to consider potential safety risks linked to BsAbs in humans, as concerns associated with possible off-target effects might be very relevant. Establishing multidisciplinary approaches to sketch a holistic path to the clinical translation of anti-CTLA-4 BsAbs will also be crucial.

Nevertheless, despite the advantages discussed above, there are still several challenges and obstacles, such as immunogenicity and chain mispairing issues in the commercial production and development of anti-CTLA-4 BsAbs. More specifically, it takes time and funds to manufacture BsAbs. Obtaining the necessary products necessitates using suitable, secure, and economical cell line manufacturing processes and analytical and purifying techniques (76). Furthermore, before patients may benefit from anti-CTLA-4 BsAbs, some post-antibody production problems, such as degradation, aggregation, denaturation, fragmentation, and oxidation of BsAbs, must be addressed (76). Further clinical trials are necessary to investigate the most effective dose and mode of administration that can result in controlled release formulations, lower systemic adverse effects, and greater concentrations in target tissues. Besides, the unavoidable negative consequences on healthy organs such as neurotoxicity or other intricate aspects, such as an immunotolerant cancer stroma, disrupted neovasculature, and insufficient penetration of BsAbs, make BsAbs targeting solid tumors deserving of additional study (77, 78). Notably, the suppressive TME, which restricts T cell activation and causes immunological insufficiency (79), is a significant barrier to anti-CTLA-4 BsAbs in advanced solid tumors. Thus, there is a genuine eagerness for the continuing investigations of anti-CTLA-4 BsAbs in solid tumors, which are predicted to provide positive outcomes soon, even if developing these BsAbs from bench to bedside may take a long time and involve a huge endeavor.

## Author contributions

PF and A-AD: conceptualization. PF and SM: writing the original draft. A-AD and MA: editing. PF: visualization. A-AD and MA: supervision. All authors contributed to the article and approved the submitted version.
